# Data-Driven Identification of Potentially Successful Intervention Implementations Using 5 Years of Opioid Prescribing Data: Retrospective Database Study

**DOI:** 10.2196/51323

**Published:** 2024-06-05

**Authors:** Lisa EM Hopcroft, Helen J Curtis, Richard Croker, Felix Pretis, Peter Inglesby, David Evans, Sebastian Bacon, Ben Goldacre, Alex J Walker, Brian MacKenna

**Affiliations:** 1 Nuffield Department of Primary Care Health Sciences University of Oxford Oxford United Kingdom; 2 Department of Economics University of Victoria Victoria, BC Canada

**Keywords:** electronic health records, primary care, general practice, opioid analgesics, data science, implementation science, data-driven, identification, intervention, implementations, proof of concept, opioid, unbiased, prescribing data, analysis tool

## Abstract

**Background:**

We have previously demonstrated that opioid prescribing increased by 127% between 1998 and 2016. New policies aimed at tackling this increasing trend have been recommended by public health bodies, and there is some evidence that progress is being made.

**Objective:**

We sought to extend our previous work and develop a data-driven approach to identify general practices and clinical commissioning groups (CCGs) whose prescribing data suggest that interventions to reduce the prescribing of opioids may have been successfully implemented.

**Methods:**

We analyzed 5 years of prescribing data (December 2014 to November 2019) for 3 opioid prescribing measures—total opioid prescribing as oral morphine equivalent per 1000 registered population, the number of high-dose opioids prescribed per 1000 registered population, and the number of high-dose opioids as a percentage of total opioids prescribed. Using a data-driven approach, we applied a modified version of our change detection Python library to identify reductions in these measures over time, which may be consistent with the successful implementation of an intervention to reduce opioid prescribing. This analysis was carried out for general practices and CCGs, and organizations were ranked according to the change in prescribing rate.

**Results:**

We identified a reduction in total opioid prescribing in 94 (49.2%) out of 191 CCGs, with a median reduction of 15.1 (IQR 11.8-18.7; range 9.0-32.8) in total oral morphine equivalence per 1000 patients. We present data for the 3 CCGs and practices demonstrating the biggest reduction in opioid prescribing for each of the 3 opioid prescribing measures. We observed a 40% proportional drop (8.9% absolute reduction) in the regular prescribing of high-dose opioids (measured as a percentage of regular opioids) in the highest-ranked CCG (North Tyneside); a 99% drop in this same measure was found in several practices (44%-95% absolute reduction). Decile plots demonstrate that CCGs exhibiting large reductions in opioid prescribing do so via slow and gradual reductions over a long period of time (typically over a period of 2 years); in contrast, practices exhibiting large reductions do so rapidly over a much shorter period of time.

**Conclusions:**

By applying 1 of our existing analysis tools to a national data set, we were able to identify rapid and maintained changes in opioid prescribing within practices and CCGs and rank organizations by the magnitude of reduction. Highly ranked organizations are candidates for further qualitative research into intervention design and implementation.

## Introduction

The prescription of opioids is common and appropriate in the management of acute pain, but their efficacy with regards to chronic pain is not supported by empirical evidence [1], and there is a global problem with opioid overuse [2]. Long-term use of opioids has been shown to be associated with the accumulating risk of dependence and overdose [3]. The continually rising rates of opioid prescription, particularly in England and Wales [4-6], prompted the publication of new guidance in 2010 [7] advocating for a cautious approach in the long-term prescribing of opioids [8], and opioids have been a specific priority for governmental advisory groups [9]. In 2019, Public Health England (PHE) published the *Prescribed Medicines Review*, which aimed to “identify the scale, distribution and causes of prescription drug dependence, and what might be done to address it” [10]. This review included data from the National Health Service Business Services Authority (NHSBSA) primary care prescription data set, which suggested that some progress had been made in reducing opioid prescribing, with a small but consistent fall in rates between 2015 and 2018. However, there was also evidence that opioid prescribing remains a persistent public health problem in England, with higher rates of prescription in areas of higher deprivation and evidence that long-term prescribing was associated with opioid overdose and dependence. The first recommendation of this report was “increasing the availability and use of data on the prescribing of medicines that can cause dependence or withdrawal to support greater transparency and accountability and help ensure practice is consistent and in line with guidance” [10].

Our group produces OpenPrescribing [11], which allows open access to the same NHSBSA primary care prescription data set used in the PHE review. It is a free and widely used tool with 20,000 unique users per month, where anyone can explore the prescriptions dispensed at any practice in England and monitor prescribing patterns down to the level of individual brands, formulations, and doses.

In OpenPrescribing, we perform automated analyses to generate monthly reports covering 80 measures of prescribing safety, effectiveness, and cost. Our analyses included all general practices (GPs) and their regional organizations, which were known as clinical commissioning groups (CCGs) at the time of this study. Several measures have been developed to capture trends and variations in opioid prescribing [12]. This window into national opioid prescribing data presents an opportunity to identify changes—both increases and decreases—in prescribing that could inform National Health Service (NHS) decision-making and policy.

It is our experience that the best practice is typically defined by organizations identifying themselves as having improved, following the implementation and internal assessment of interventions. We are seeking to pursue an alternative, data-driven, and unbiased approach that instead exploits the national prescribing data set to identify prescribing patterns that may be representative of best practice (ie, where we can identify a significant reduction in opioid prescribing).

We set out to apply our change detection algorithm [13] to identify patterns indicative of maintained and significant reduction that may help identify best practices with regard to opioid prescribing policy.

## Methods

### Study Design

We conducted a retrospective database study using GP primary care electronic health record data from all GPs in England.

### Data Source

We extracted data from the OpenPrescribing database. This imports openly accessible prescribing data from the large monthly files published by the NHSBSA, which contain data on cost and items prescribed for each month, for every typical GP and CCG in England since mid-2010 [14]. We extracted data up to November 2019. We note that CCGs were replaced by integrated care boards as of July 1, 2022. We have retained results by CCGs as this was an active administrative unit of the NHS in England during the study period. The monthly prescribing data sets contain 1 row for each different medication and dose, in each prescribing organization in NHS primary care in England, describing the number of items (ie, prescriptions issued) and the total cost. These data are sourced from community pharmacy claims data and, therefore, contain all items that were dispensed. We extracted all available data for typical GPs, excluding other organizations such as prisons and hospitals, according to the NHS Digital data set of practice characteristics [15]. The numbers of patients registered at each practice were obtained from NHS Digital [15].

### Study Measures

A total of 3 measures were used in this study to capture various aspects of opioid prescribing. The first (“total oral morphine equivalence per 1000 patients”) expresses the oral morphine equivalence (OME) of all opioid prescriptions per 1000 patients [16]. The second and third look to capture information about regularly prescribed opioids— those used on a regular basis to control pain rather than preparations used for breakthrough pain or opioid injections. Of the regularly prescribed opioids, high-dose opioids were defined as those with ≥120 mg OME per day [8]. The “high dose opioids as percentage regular opioids” measure captures the number of prescriptions of these high-dose, regularly prescribed opioids as a percentage of all long-acting opioids [17]; the “high dose opioid items per 1000 patients” measure captures the same number of high-dose, long-acting opioids but expresses this per 1000 patients [18]. For all measures, higher values represent higher rates of opioid prescription.

In England, an individual will be registered at 1 GP or practice; and each practice belonged, at the time of analysis, to a regional CCG that can influence their prescribing. These organizations and their membership can change over time (eg, a practice may be reassigned to a different CCG, a CCG may be renamed or replaced, or a practice may close). In our results, we report results for any practice or CCG that existed during the study period, acknowledging that some of these no longer exist. CCGs have now also been replaced with subintegrated care board locations, but some still retain their previous CCG code.

Practices may act independently to change prescribing or participate in an action coordinated by their CCG. We, therefore, conducted analysis at both organizational levels. Monthly values for each measure were calculated for every practice and CCG between December 2014 and November 2019 (this study period was chosen so as to assess prescribing rates over a reasonable period of time, without being affected by the COVID-19 pandemic). The monthly data were summarized as deciles and presented as decile charts across all practices or CCGs each month.

### Statistical Methods

For this study, we used our innovative change detection Python library (available via the Python Package Index) [19], which is an automated method of detecting change in time-series data. This algorithm was originally developed to determine how clinicians vary in their response to new guidance on existing or new interventions. By measuring the timing and magnitude of change in the relevant organizations, it is able to identify both steep, sudden changes and more gradual, smooth transitions over multiple months. The full methods are described elsewhere [13] and the code is available for anyone to use as a single command with our open Python library [19].

Data for each of the 3 measures were analyzed for all 191 CCGs and 7458 practices. The time series for each organization was analyzed using our change detection algorithm (using the default parameters) to identify the location and magnitude of significant reductions in the measure (substantial increases were filtered out as they are not relevant to the research question). These results were then filtered to remove (1) a total of 678 closed or dormant practices and (2) a further 237 practices with a list size of less than 2000 (this latter group was excluded to avoid analyses of time series with a high level of noise due to low prescribing volume); this process left 6543 practices to be subject to further analysis. We filtered out practices where more than half of the monthly denominator values were 0 (either no registered population or no total opioid prescribing as per measure definitions) across the study period. Among the organizations where our code detected a substantial reduction, for each measure, we selected those whose starting level immediately before the reduction was in the top 20% of all practices (top 150) or CCGs (top 38); this was to remove any organizations with consistently low prescribing from our results. For each measure, we then ranked practices and CCGs by the total measured change (the percentage reduction between the predrop value and the end-drop value) to identify which organizations exhibited the most substantial reductions.

The decile plots provided show an individual organization’s prescribing rates across the period (thick red line), in the context of all peer organizations (summarized using deciles, as blue lines).

### Software and Reproducibility

Data management and analysis were carried out using Python (version 3.8; Python Software Foundation) and Google BigQuery. Our change detection library [19] is a Python wrapper for the *GETS* R package [20]. All our methods and underlying code are openly available on GitHub [21]. The full results, summary statistics of changes detected, and top 10 CCGs and practices can be seen in the notebooks folder, in the files *ccg-opioids-change-detection-analysis.ipynb* and *practice-opioids-change-detection-analysis.ipynb*. All organizations that existed in the study period (including those that have since closed or been replaced) are included in these reports.

### Ethical Considerations

This study uses open, publicly available, and anonymized data. This analysis did not need a review from an institutional review board because it used previously collected, fully anonymized data [22]. Informed consent and compensation were similarly not required and would not be possible.

## Results

### Overview

We identified substantial reductions in at least 49% of all CCGs (94/191, 49.2%) and practices (4100/7460, 55%) for all measures; summary statistics for these reductions are provided in Table 1. Note that these data describe all substantial reductions detected, that is, before filtering for a top 20% (top 38 CCGs or top 150 practices) starting value. For both CCGs and practices, reductions are on average greater for both high-dose opioid prescribing measures as compared to those observed for the total OME measure, although the IQR values demonstrate that there is also more variability in the high-dose opioid prescribing measures. Reductions appear more modest among CCGs than practices (with lower medians and lower maximum values), but these reductions may be more consistent (with lower variability and greater minimum values observed in CCGs as compared to practices). There is at least 1 practice in each measure where the reduction is almost 99% to 100% and at least 1 practice where the reduction detected is very close to 0.

**Table 1:** Summary of all opioid reductions identified across clinical commissioning groups (n=191) and practices (n=7458) in England between December 2014 and November 2019 using the automated change detection algorithm^a^.

Organization and measure			Count, n		Reduction (%), median (IQR)		Reduction (%), range
**Clinical commissioning groups (n=191)**							
	Total OME^b^ per 1000 patients	94		15.1 (11.8-18.7)		9.0-32.8	
	High-dose opioids as percentage regular opioids	168		19.0 (13.7-25.8)		3.6-41.5	
	High-dose opioids per 1000 patients	115		22.2 (17.2-30.0)		1.0-45.4	
**Practices (n=7460)**							
	Total OME per 1000 patients	4100		28.2 (19.8-39.7)		0.1-99.1	
	High-dose opioids as percentage regular opioids (%)	4632		47.7 (33.0-65.9)		0.0-100.0	
	High-dose opioids per 1000 patients	4334		56.0 (37.7-73.4)		0.0-100.0	

^a^Count indicates the number of organizations (clinical commissioning groups or practices) in which a reduction was identified. Median, IQR, and range summarize the size of the reductions identified in those organizations (expressed as % reduction from the predrop value to the end-drop value).

^b^OME: oral morphine equivalence.

### Changes for CCGS

Table 2 illustrates the CCGs that exhibited the biggest reduction in each of the 3 OpenPrescribing measures over the study period, detailing the proportion of change and the month in which the change started. Note that these CCGs meet the criteria for identification, that is, their prescribing rate immediately before the reduction was in the top 38 (20%) CCGs.

The total OME measure shows a gradual reduction over time in all 3 CCGs, with the algorithm identifying a reduction of up to 31%. The results for the 2 regular high-dose opioid measures also exhibit a gradual reduction over time but capture greater reductions in regular high-dose opioid prescription, with 40% and 39% reductions identified as a proportion of all regular opioids and per 1000 patients respectively.

**Table 2:** Automatically detected changes across 3 measures of opioid prescribing in a retrospective prescribing database study of CCGs^a^ in England^b^.

Measure and rank		CCG	Absolute change detected (difference)^c^	Proportional change (%)^d^	Month when change was detected	Decile chart
**Total OME^e^ per 1000 patients**						
	1	Vale Royal	15,461	31	November 2015	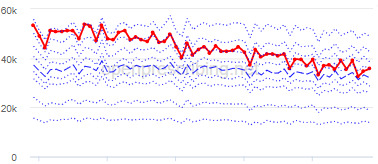
	2	Great Yarmouth and Waveney	19,182	26	February 2017	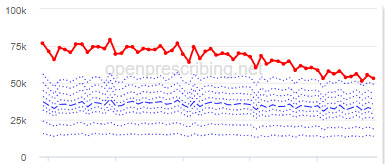
	3	Heywood, Middleton and Rochdale	15,393	26	August 2017	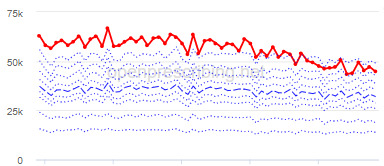
**High-dose opioids as percentage regular opioids^f^**						
	1	North Tyneside	8.9	40	September 2018	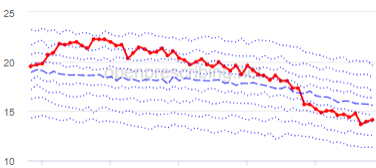
	2	Great Yarmouth and Waveney	8.7	33	May 2018	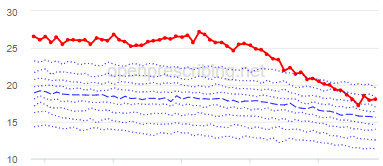
	3	Heywood, Middleton and Rochdale	8.9	33	September 2018	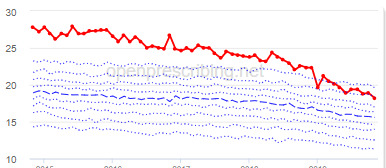
**High-dose opioid items per 1000 patients**						
	1	Great Yarmouth and Waveney	2.0	39	August 2017	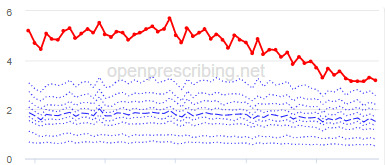
	2	Hastings and Rother	1.3	39	February 2018	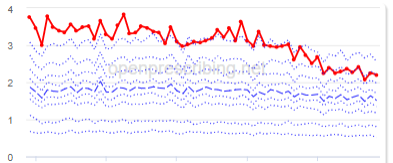
	3	Heywood, Middleton and Rochdale	1.6	38	August 2017	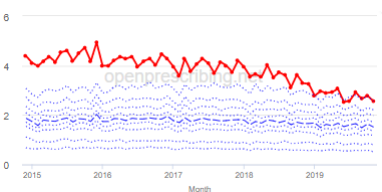

^a^CCG: clinical commissioning group.

^b^Ranked top 3 CCGs exhibiting a reduction in each of the OpenPrescribing opioid measures (December 2014 to November 2019). The decile chart shows the prescription rate for the CCG as a thick red line; prescribing rates for all other CCGs are summarized using deciles (dotted blue lines) with the median highlighted (thick dashed blue line). Note, y-axis scales differ.

^c^The absolute change is the difference between the starting value and final value during the detected change period.

^d^The relative change gives the difference as a percentage of the starting value.

^e^OME: oral morphine equivalence.

^f^This measure is calculated as a percentage, so the absolute change represents the percentage points difference.

### Changes for Practices

Table 3 illustrates the practices that exhibited the biggest change in each of the 3 OpenPrescribing measures over the study period, detailing the proportion of change and the month in which the change started. Note that these practices meet the criteria for identification as described in the “Statistical Methods” section, that is, their prescribing rate immediately before the reduction was in the top 150 (20%) practices.

The practice time series (Table 3) are noticeably different from those of the CCGs (Table 2), the magnitude of the measured changes is larger, and the slope of the time series is much steeper for practices. In the case of the regular high-dose opioids as a percentage of all opioids, all 3 practices are seen to completely eliminate all regular high-dose opioids for several months; similarly, very low values are observed for the top 3 practices with regards to reductions in high-dose opioid items per 1000 patients.

**Table 3:** Automatically detected changes across 3 measures of opioid prescribing in a retrospective prescribing database study of practices in England^a^.

Measure and rank		Practice	Absolute change detected (difference)^b^	Proportional change (%)^c^	Month when change was detected	Chart
**Total OME^d^ per 1000 patients**						
	1	Practice A (Manchester CCG^e^)	76,161	74	June 2018	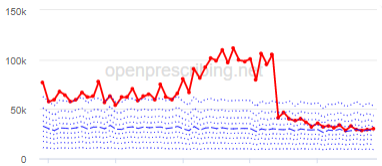
	2	Practice B (Manchester CCG)	38,574	62	September 2018	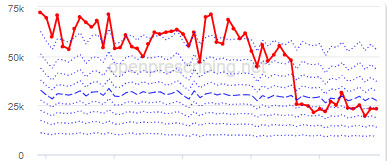
	3	Practice C (West Cheshire CCG)	88,109	61	February 2017	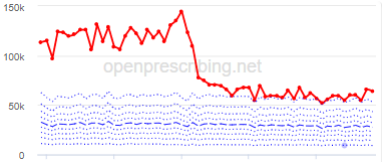
**High-dose opioids as percentage regular opioids^f^**						
	1	Practice D (City and Hackney CCG)	44	99	August 2018	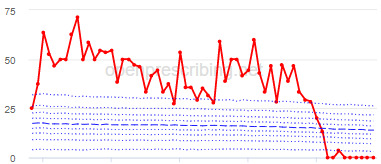
	2	Practice E (Harrow CCG)	52	99	May 2017	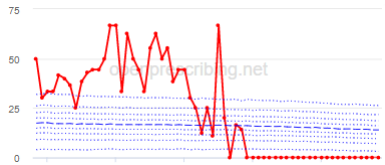
	3	Practice F (Ealing CCG)	95	99	March 2016	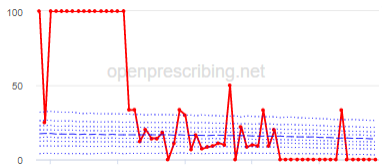
**High-dose opioid items per 1000 patients**						
	1	Practice G (Portsmouth CCG)	3.6	97	August 2018	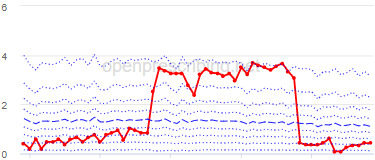
	2	Practice H (Coventry and Rugby CCG)	4.8	97	February 2018	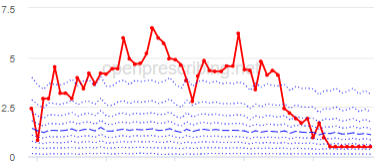
	3	Practice I (Salford CCG)	4.8	95	February 2018	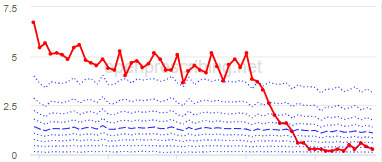

^a^Ranked top 3 practices exhibiting a reduction in each of the OpenPrescribing opioid measures (December 2014 to November 2019). The decile chart shows the prescription rate for the practice as a thick red line; prescribing rates for all other practices are summarized using deciles (dotted blue lines) with the median highlighted (thick dashed blue line). Note, y-axis scales differ*.*

^b^The absolute change is the difference between the starting value and final value during the detected change period.

^c^The relative change gives the difference as a percentage of the starting value.

^d^OME: oral morphine equivalence.

^e^CCG: clinical commissioning group.

^f^This measure is calculated as a percentage, so the absolute change represents the percentage points difference.

## Discussion

### Main Findings

We have identified significant reductions in 3 measures of opioid prescribing using a data-driven approach in over 7000 practices across 191 CCGs in England (Table 1). These organizations have then been ranked by the magnitude of reduction to identify where the largest reductions have been realized. The top-ranked CCGs exhibit a slow and gradual reduction in opioid use (Tables 1 and 2); by contrast, the top-ranked practices exhibit rapid and sudden reductions over a few months (Tables 1 and 3). Opioid prescribing and treatment of pain more broadly can be complex, but our findings illustrate that some CCGs and practices appear to have significantly reduced their prescribing of opioids over the study period, more so than many of their peers.

### Findings in Context

The PHE review identified evidence of tentative progress in reducing opioid prescribing between 2015 or 2016 and 2017 or 2018 [10]. Our analysis includes and extends this time period and finds evidence that some organizations may be driving this tentative progress more than others (eg, the CCGs reported in Table 2).

We do have evidence that 1 of the organizations that has emerged as a potential candidate by our methodology is a genuine example of improved performance. Between 2017 and 2019, Great Yarmouth and Waveney designed and implemented an extensive program of opioid reduction interventions, including target trajectories for improvement; incentive schemes for clinicians; dialogue with practice pharmacists, patient groups, and relevant clinical groups (eg, prescribing leads and pain management teams); new patient information materials; collecting case studies for discussion; and associated press and social media to raise awareness. While this CCG still exhibits high levels of opioid prescribing, rates have reduced significantly, with the organization being recognized for this progress nationally [23]. Our methodology ranked Great Yarmouth and Waveney as first (reduction of 39% [absolute reduction 2.0] starting in August 2017) for high-dose opioid prescribing per 1000 patients and second (reduction of 33% [absolute reduction 8.7%] starting in May 2018) for high-dose opioid prescribing as a percentage of regular opioid prescribing, aligning with the period of intervention implementation.

The new national policy for the optimization and personalization of various addiction-forming medications, including opioids, lacks practical detail on implementation for GPs to reduce opioid prescribing [24,25]. Different innovations are being implemented in the United Kingdom [26-28], and these strategies are associated with observable changes in prescribing practices. However, some may succeed in 1 area but not another. GPs undertake complex decision-making on opioid prescribing, balancing benefits and harms [29], but struggle with limited time and alternatives for chronic pain [30,31]. Further practical guidelines for GPs on appropriate prescribing could help with tapering and effective communication strategies [26,30]. Tools such as that demonstrated in this paper, highlighting positive changes, could help to inspire and motivate practices or regions to make changes, while also giving them other organizations to contact about how changes have been achieved.

### Implications for Research and Policy

We are seeking to implement this methodology as a new “Improvement Radar” tool on OpenPrescribing, with the intention of systematically identifying candidates for further qualitative research across multiple important public health prescribing measures to better understand the patterns shown, for example, to uncover and learn from effective practice or refine our measures to exclude artifacts. It is our experience that best practice is typically defined by organizations identifying themselves as having improved, following implementation and internal assessment of interventions. Using the Improvement Radar, policy makers interested in spreading the best practice can systematically identify organizations that may have already implemented effective interventions. However, it is critical that policy makers undertake further investigations for reasons outlined in the limitations. This tool offers an opportunity to reduce the resources needed to identify best practices. Similarly, local medicines optimization teams may wish to use data and tools like this to identify peers across the country who have already delivered successful interventions to inform local initiatives. Further quantitative research is also possible from the data set, for example, drilling down by opioid type and monitoring the impact of any local or national interventions or policies.

### Strengths and Limitations

The national prescribing data used for this analysis, being collected for reimbursement purposes, are highly complete and accurate. We have taken into account most (6543/7458, 87.7%) typical primary care practices in England, thereby minimizing the risk of biased sampling. Executing this analysis in an existing, open platform such as OpenPrescribing ensures accountability and transparency—both identified as priorities in the PHE report [10]; by default, all code in this study, from data curation to completed output, is shared openly on GitHub and the Python Package Index. Furthermore, there exists a robust and tested framework with which relevant new measures can be introduced or existing measures can be amended as required in order to respond to any evolving change in tackling opioid dependency and abuse. Our use of OME conversion permits the reporting of trends for opioid medicines overall while accounting for variation in strength.

We also note some limitations. First, the prescribing data set does not include secondary care prescriptions as this was unavailable at the time of the study [32], and as such, the opioid measures implemented here may underestimate the extent of opioid prescribing nationally, although financial data would indicate that the vast majority of analgesics (British National Formulary (BNF) section 4.7, which includes the BNF subsection 4.7.2 opioid analgesics) are prescribed in primary care [33]. Second, we acknowledge that practice-level time-series data, in particular, could be significantly impacted by local circumstances, including low patient numbers, a change in patient population, a change to prescription frequency (eg, from weekly to monthly scripts), or a shift in responsibility of opioid prescribing (eg, from primary to secondary care) and, therefore, that an apparent reduction in any opioid measure may not be due to a successful intervention. For example, practice G (Table 3) rapidly increased their high-dose opioid items per 1000 patients in 2016 followed by a similar rapid reduction 2 years later; this could be due to a change to daily prescribing as can be clinically justified for some patients. While we acknowledge these limitations, it is important to note that the intention of this methodology was always to rank or prioritize organizations for further investigation, rather than definitively ascribe reductions in opioid prescribing to successful interventions.

### Conclusions

We have demonstrated that data-driven approaches to detect substantial changes in time-series data have potential value in the context of opioid prescribing. We have been able to rank organizations with regards to the extent of opioid prescribing reduction; organizations occupying the top of that list show large drops that warrant further qualitative investigation and could be indicative of success in tackling an important public health concern.

Should this further qualitative research reveal that reductions have been driven by well-designed and well-implemented interventions, methods of best practice will have been identified using an unbiased, evidence-based approach. The organizations found to be implementing this best practice may have valuable insights, approaches, and policies to share regarding how positive change can be achieved elsewhere. It also demonstrates, particularly in the most robust and gradual change observed among CCGs, that positive change is possible and, therefore, that continued and wider success in reducing opioid prescribing is dependent, at least in part, on closing the implementation gap.

## Abbreviations

BNF: British National Formulary
CCG: clinical commissioning group
GP: general practice
NHS: National Health Service
NHSBSA: National Health Service Business Services Authority
OME: oral morphine equivalence
PHE: Public Health England

## Data Availability

The data sets generated and analyzed during this study are available in the “opioids-change-detection-notebook” repository [21]. All our methods and code can also be found in this repository [21]. The underlying data are available via the National Health Service Business Services Authority (NHSBSA) [14].
